# Distinct Action of Flavonoids, Myricetin and Quercetin, on Epithelial Cl^−^ Secretion: Useful Tools as Regulators of Cl^−^ Secretion

**DOI:** 10.1155/2014/902735

**Published:** 2014-04-10

**Authors:** Hongxin Sun, Naomi Niisato, Kyosuke Nishio, Kirk L. Hamilton, Yoshinori Marunaka

**Affiliations:** ^1^Department of Molecular Cell Physiology Graduate School of Medical Science, Kyoto Prefectural University of Medicine, Kyoto 602-8566, Japan; ^2^Japan Institute for Food Education and Health, St. Agnes' University, Kyoto 602-8013, Japan; ^3^Shin-Koiwa Clinic, Tokyo 124-0023, Japan; ^4^Department of Physiology, School of Medical Sciences, University of Otago, Dunedin 9054, New Zealand; ^5^Department of Bio-Ionomics, Graduate School of Medical Science, Kyoto Prefectural University of Medicine, Kyoto 602-8566, Japan

## Abstract

Epithelial Cl^−^ secretion plays important roles in water secretion preventing bacterial/viral infection and regulation of body fluid. We previously suggested that quercetin would be a useful compound for maintaining epithelial Cl^−^ secretion at a moderate level irrespective of cAMP-induced stimulation. However, we need a compound that stimulates epithelial Cl^−^ secretion even under cAMP-stimulated conditions, since in some cases epithelial Cl^−^ secretion is not large enough even under cAMP-stimulated conditions. We demonstrated that quercetin and myricetin, flavonoids, stimulated epithelial Cl^−^ secretion under basal conditions in epithelial A6 cells. We used forskolin, which activates adenylyl cyclase increasing cytosolic cAMP concentrations, to study the effects of quercetin and myricetin on cAMP-stimulated epithelial Cl^−^ secretion. In the presence of forskolin, quercetin diminished epithelial Cl^−^ secretion to a level similar to that with quercetin alone without forskolin. Conversely, myricetin further stimulated epithelial Cl^−^ secretion even under forskolin-stimulated conditions. This suggests that the action of myricetin is via a cAMP-independent pathway. Therefore, myricetin may be a potentially useful compound to increase epithelial Cl^−^ secretion under cAMP-stimulated conditions. In conclusion, myricetin would be a useful compound for prevention from bacterial/viral infection even under conditions that the amount of water secretion driven by cAMP-stimulated epithelial Cl^−^ secretion is insufficient.

## 1. Introduction

Water secretion across epithelial tissues contributes to prevention of our body from bacterial/viral infection and regulation of body fluid content. The water secretion across epithelial tissues is driven by epithelial Cl^−^ secretion [1–3]. The epithelial Cl^−^ secretion consists of two steps: (1) the Cl^−^-entry step across the basolateral membrane via Cl^−^ transporter participating in Cl^−^ uptake into the intracellular space such as Na^+^-K^+^-2Cl^−^ cotransporter (NKCC) and (2) the Cl^−^-releasing step across the apical membrane via Cl^−^ channels such as cystic fibrosis transmembrane conductance regulator (CFTR) Cl^−^ channel at the apical membrane [4, 5]. The continuous Cl^−^ secretion requires stimulation of Cl^−^ uptake transporter such as NKCC [6, 7]. Flavonoids and flavonoid-like compounds have various actions in cell function [8, 9]. Our previous studies have reported that a flavonoid, quercetin, elevates epithelial Cl^−^ secretion under basal conditions, but decreases it under cAMP-stimulated conditions [10] by modifying activity of NKCC [3]. These reports suggest that quercetin regulates the activity of NKCC leading epithelial Cl^−^ secretion to a moderate level irrespective of cAMP-induced stimulation and that quercetin would be a useful compound to achieve a moderate level of epithelial Cl^−^ secretion. However, in some cases, cAMP-induced stimulation is not large enough to maintain an adequate level of epithelial Cl^−^ secretion. Myricetin has been reported to demonstrate promotive and protective effects on intestinal tight junctional barriers of epithelia and it has antiviral function [11–13]. Therefore, it would be important to identify a useful compound with stimulatory actions on epithelial Cl^−^ secretion even under cAMP-stimulated conditions. Our study examines the effects of quercetin and myricetin on Cl^−^ secretion in the absence and presence of cAMP-stimulation of renal A6 cells.

## 2. Materials and Methods

### 2.1. Chemicals and Materials

**Figure 1:**
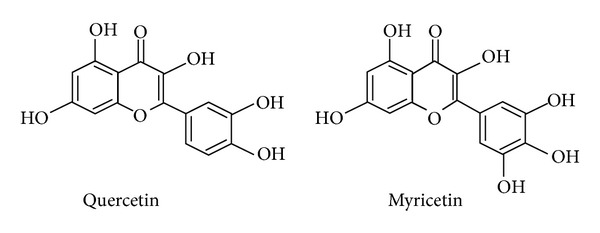
Structures of quercetin and myricetin.

We obtained forskolin, benzamil, NPPB (5-nitro-2-(3-phenylpropylamino)benzoic acid), quercetin, myricetin, and dimethyl sulfoxide (DMSO) from Sigma-Aldrich (St. Louis, MO, USA) and epithelial A6 cells from American Type Culture Collection (ATCC). Forskolin, benzamil, NPPB, bumetanide, quercetin, and myricetin were dissolved in DMSO. We applied forskolin of 10 *μ*M, benzamil of 10 *μ*M, NPPB of 100 *μ*M, bumetanide of 100 *μ*M, quercetin of 100 *μ*M, and myricetin of 100 *μ*M to the bath solution as the final concentration. The concentrations of forskolin, quercetin and myricetin used in the present study were determined from the observations obtained in previous reports [7, 10]. The structures of quercetin and myricetin are shown in Figure 1.

### 2.2. Cell Culture

Renal epithelial A6 cells derived from* Xenopus laevis* were obtained from American Type Culture Collection (Rockville, MD, USA) at* passage 68*. We cultured A6 cells (*passages 73–84*) on plastic flasks at 27°C in a humidified incubator with 1.0% CO_2_ in air in a culture medium containing 75% (vol/vol) NCTC-109, 15% (vol/vol) distilled water and 10% (vol/vol) fetal bovine serum. Then, we seeded cells onto permeable tissue culture-treated Transwell filter cups (Costar, Cambridge, MA, USA) for electrophysiological measurements at a density of 5 × 10^4^ cells/well for 11–15 days.

### 2.3. Measurement of Transepithelial Conductance (Gt)

We transferred monolayers of A6 cells subcultured on tissue culture-treated Transwell filter cups to a modified Ussing chamber (Jim's Instrument, Iowa City, IA, USA) designed to hold the filter cup and continuously measured transepithelial potential difference (PD) by a high-impedance millivoltmeter (VCC-600, Physiologic Instrument, San Diego, CA, USA) [7]. We applied a pulse of 1 *μ*A constant current every 10 s for 0.5 s to the A6 monolayer under open-circuit conditions. This enabled us to calculate the transepithelial conductance (Gt) from the change in the PD (ΔPD) caused by the 1 *μ*A constant-current pulse using Ohm's law (Gt = 1 *μ*A/ΔPD mV). We applied 100 *μ*M NPPB (a nonselective Cl^−^ channel blocker [3, 14, 15]) to the apical solution for detection of NPPB-sensitive conductance. We measured an NPPB-sensitive conductance by calculating the difference between the Gt just before and 30 minutes after application of 100 *μ*M NPPB. This difference of Gt represents an NPPB-sensitive conductance. In the present study, we use the NPPB-sensitive conductance as the apical Cl^−^ conductance. The NPPB-sensitive conductance indicates the apical Cl^−^ channel conductance, since the apical Cl^−^ conductance is much smaller than the basolateral Cl^−^ conductance [16]. Further, apical application of 100 *μ*M NPPB diminishes the apical Cl^−^ conductance but not the basolateral Cl^−^ conductance [7, 16]. Bumetanide has been shown to have no effects on the NPPB-sensitive conductance [7]. These observations [7, 16] indicate that the NPPB-sensitive conductance can be used as the apical Cl^−^ conductance.

### 2.4. Measurement of Short-Circuit Current (Isc)

As reported previously [7, 16], we measured a short-circuit current (Isc) in A6 cells. The Isc measured directly by clamping the PD to 0 mV was identical to the calculated current as Gt*·*PD (equivalent current); namely, the monolayer had a linear current-voltage relationship. In the present study, we show an equivalent current (Gt*·*PD) as Isc. A positive current represents a net flow of anions from the basolateral solution to the apical one [7].

### 2.5. Solutions

The solution used in the present study contained 120 mM NaCl, 3.5 mM KCl, 1 mM CaCl_2_, 1 mM MgCl_2_, 5 mM glucose, and 10 mM HEPES with pH 7.4.

### 2.6. Temperature

All experiments shown in the present study were performed at 24-25°C.

### 2.7. Data Presentation

Values of Isc and Gt are shown as the mean, and the error bar indicates SEM. ANOVA was used for statistical analysis, and *P* < 0.05 was considered significant.

## 3. Results

### 3.1. Effects of Quercetin and Myricetin on Isc under Basal Conditions

**Figure 2:**
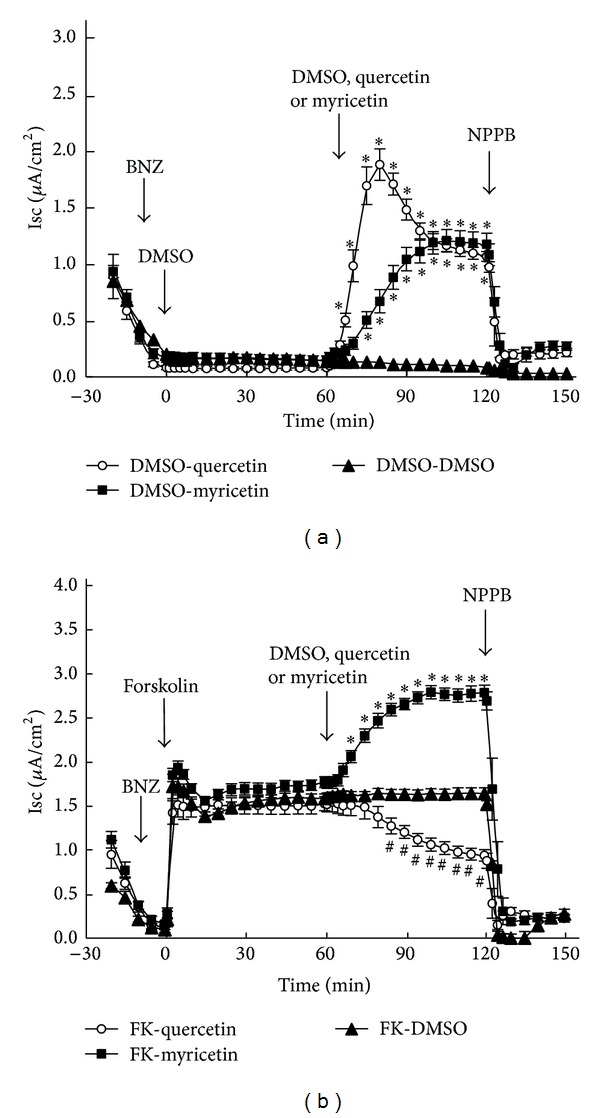
Effects of quercetin and myricetin on Isc under basal (a) and forskolin-stimulated conditions (b). (a) Benzamil (BNZ, a blocker of epithelial Na^+^ channel: ENaC; 10 *μ*M) was applied to the apical solution at −10 min. DMSO (dimethyl sulfoxide; a solvent for forskolin; 0.1% as the final concentration in Isc measuring solutions) was added to both apical and basolateral solutions at 0 min (open circles, closed squares, and closed triangles). Quercetin (100 *μ*M; open circles), myricetin (100 *μ*M; closed squares), or DMSO (a solvent for quercetin and myricetin; 0.1%; closed triangles) was applied to both apical and basolateral solutions at 60 min. NPPB (a nonspecific blocker of Cl^−^ channels; 100 *μ*M) was applied to the apical solution at 120 min (open circles, closed squares, and closed triangles). *n* = 5 for DMSO, *n* = 6 for quercetin, and *n* = 7 for myricetin. (b) Benzamil (BNZ, 10 *μ*M) was applied to the apical solution at −10 min. Forskolin (10 *μ*M) was added to both apical and basolateral solutions at 0 min (open circles, closed squares, and closed triangles). Quercetin (100 *μ*M; open circles), myricetin (100 *μ*M; closed squares), or DMSO (a solvent for quercetin and myricetin; 0.1%; closed triangles) was applied to both apical and basolateral solutions at 60 min. NPPB (100 *μ*M) was applied to the apical solution at 120 min (open circles, closed squares and closed triangles). *n* = 6 for DMSO, *n* = 5 for quercetin, and *n* = 8 for myricetin. The values marked with ∗ (open circles and closed squares) are significantly larger than DMSO (closed triangles; *P* < 0.05). The values marked with # (closed squares) are significantly smaller than DMSO (closed triangles; *P* < 0.05).

A6 cells can exhibit Cl^−^ secretion and Na^+^ absorption [7, 17–22]. Since the purpose of the present study was to examine transport properties of Cl^−^ secretion, we added benzamil (10 *μ*M) to the apical solution to block Na^+^ absorption via epithelial Na^+^ channel (ENaC) in A6 cells (Figure 2). We used 10 *μ*M benzamil, because it has been demonstrated to completely block ENaC activity [18, 23, 24]. DMSO (a solvent control for quercetin and myricetin (Figure 1)) applied to both apical and basolateral solutions had no effect on Isc (closed triangles as in Figure 2(a)), and 100 *μ*M NPPB applied to the apical solution abolished the benzamil-insensitive Isc (*i.e.*, the residual Isc after application of benzamil was abolished) (Figure 2(a)). Quercetin significantly stimulated Isc (open circles as in Figure 2(a)), and NPPB abolished the quercetin-stimulated Isc (open circles as in Figure 2(a)) suggesting that quercetin stimulated a Cl^−^-dependent Isc. Myricetin also elevated Isc (closed squares as in Figure 2(a)), and the myricetin-elevated Isc was sensitive to NPPB (closed squares as in Figure 2(a)). Quercetin or myricetin showed no effects on Isc in A6 cells pretreated with NPPB (data not shown). These observations indicate that quercetin and myricetin stimulate the Cl^−^ secretion.

### 3.2. Effects of Quercetin and Myricetin on Isc under Forskolin-Stimulated Conditions

In order to examine the effects of quercetin and myricetin in the presence of elevated cAMP levels, we used forskolin to activate adenylyl cyclase to increase cellular cAMP. As shown in Figure 2(a) (closed triangles), DMSO, a solvent control for forskolin, had no effect on Isc. Though, forskolin stimulated Isc (open circles, closed squares, and closed triangles in Figure 2(b)), but not in the presence of NPPB (data not shown), suggesting that forskolin stimulated an NPPB-sensitive Isc. Under forskolin-stimulated conditions, DMSO (a solvent control for quercetin and myricetin) had no effect on Isc (closed triangles as in Figure 2(b)). However, in the presence of forskolin, quercetin significantly diminished Isc (open circles as in Figure 2(b)) unlike that observed during basal conditions (open circles as in Figure 2(a)). On the other hand, myricetin significantly stimulated Isc (closed squares as in Figure 2(b)) in the presence of forskolin similar to that under basal conditions (closed squares as in Figure 2(a)). Further, we applied bumetanide (a blocker of NKCC) to study the Isc observed in the present study. Application of bumetanide (100 *μ*M) almost completely diminished the Isc irrespective of the presence of forskolin, quercetin, or myricetin without any effects on Gt: the Isc in the presence of bumetanide was reduced to ~0.2 *μ*A/cm^2^ irrespective of the presence of forskolin, quercetin, or myricetin. Further, the presence of bumetanide did not significantly influence effects of forskolin, quercetin, or myricetin on Gt (data not shown). Thus, these observations indicate that the Isc observed in the present study is mediated by NKCC irrespective of the presence of forskolin, quercetin, or myricetin.

### 3.3. NPPB-Sensitive Isc under Basal and Forskolin-Stimulated Conditions

**Figure 3:**
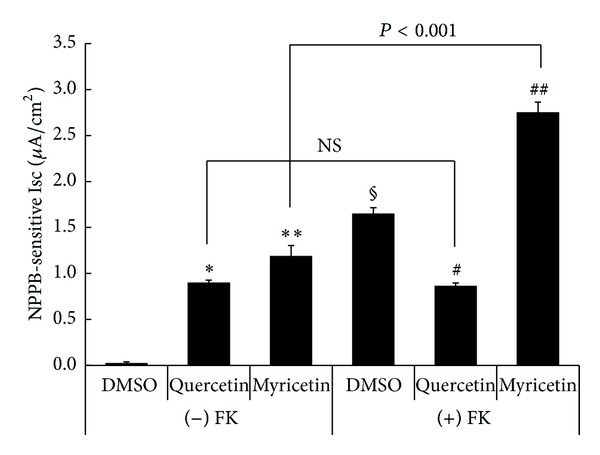
NPPB-sensitive Isc. The NPPB-sensitive Isc was measured as the difference of Isc just before and 30 min after addition of 100 *μ*M NPPB to the apical solution. *n* = 5 for DMSO, *n* = 6 for quercetin, and *n* = 7 for myricetin without forskolin ((−) FK). *n* = 6 for DMSO, *n* = 5 for quercetin, and *n* = 8 for myricetin with forskolin ((+) FK). Under basal conditions ((−) FK), the values of quercetin-stimulated Isc (∗) and myricetin-stimulated NPPB-sensitive Isc (∗∗) were significantly larger than the NPPB-sensitive Isc with DMSO alone (solvent control; *P* < 0.001). The value of Isc with DMSO alone under forskolin-stimulated conditions (DMSO marked with § in (+) FK) was significantly larger than that with DMSO alone under basal conditions (DMSO in (−) FK; *P* < 0.001). Under forskolin-stimulated conditions ((+) FK), the value of quercetin-stimulated Isc (Quercetin in (+) FK marked with #) was significantly smaller than that with DMSO alone (DMSO in (+) FK; *P* < 0.001). On the one hand, under forskolin-stimulated conditions ((+) FK), the value of myricetin-stimulated Isc (##) was significantly larger than that with DMSO alone (DMSO in (+) FK; *P* < 0.001). The value of quercetin-stimulated Isc was identical irrespective of forskolin stimulation (Quercetin in (−) FK versus Quercetin in (+) FK; NS, no significant difference), while the value of myricetin-stimulated Isc under forskolin-stimulated conditions (Myricetin in (+) FK) was significantly larger than that under basal condition (Myricetin in (−) FK; *P* < 0.001).

**Figure 4:**
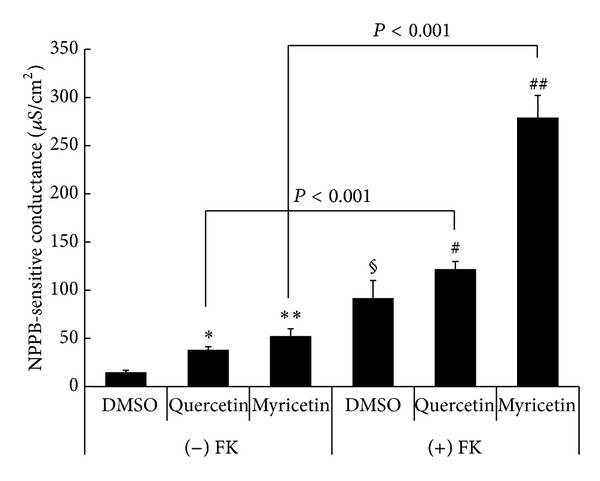
NPPB-sensitive conductance. The NPPB-sensitive conductance was measured as the difference of Isc just before and 30 min after addition of 100 *μ*M NPPB to the apical solution. *n* = 4 for DMSO, *n* = 7 for quercetin, and *n* = 11 for myricetin without forskolin ((−) FK). *n* = 11 for DMSO, *n* = 7 for quercetin, and *n* = 10 for myricetin with forskolin ((+) FK). Under basal conditions ((−) FK), the values of quercetin-stimulated conductance (∗) and myricetin-stimulated NPPB-sensitive conductance (∗∗) were significantly larger than the NPPB-sensitive conductance with DMSO alone (solvent control; *P* < 0.001). The value of Isc with DMSO alone under forskolin-stimulated conditions (DMSO marked with § in (+) FK) was significantly larger than that with DMSO alone under basal conditions (DMSO in (−) FK; *P* < 0.005). Under forskolin-stimulated conditions ((+) FK), the value of quercetin-stimulated Isc (Quercetin in (+) FK marked with #) was slightly but significantly larger than that with DMSO alone (DMSO in (+) FK; *P* < 0.05). Further, under forskolin-stimulated conditions ((+) FK), the value of myricetin-stimulated Isc (##) was significantly larger than that with DMSO alone (DMSO in (+) FK; *P* < 0.001). The quercetin-stimulated NPPB-sensitive conductance under forskolin-stimulated conditions (Quercetin in (+) FK) was significantly larger than that under basal condition (Quercetin in (−) FK; *P* < 0.001). The myricetin-stimulated NPPB-sensitive conductance under forskolin-stimulated conditions (Myricetin in (+) FK) was significantly larger than that under basal condition (Myricetin in (−) FK; *P* < 0.001).

In Figure 3, we show the NPPB-sensitive Isc under various experimental and control conditions in the absence and presence of forskolin-stimulated Isc as shown in Figure 2. Under basal conditions, quercetin increased the NPPB-sensitive Isc (*P < 0.001 compared with DMSO as in Figure 3) and myricetin also increased the NPPB-sensitive Isc (**P < 0.001 compared with DMSO as in Figure 3). Quercetin and myricetin had similar effects on the NPPB-sensitive Isc under basal conditions ((−) FK, Figure 3), although the quercetin-stimulated NPPB-sensitive Isc (0.90 ± 0.03 *μ*A/cm^2^) was slightly smaller than the myricetin-stimulated one (1.18 ± 0.12 *μ*A/cm^2^; *P* < 0.05). The NPPB-sensitive Isc in forskolin-treated cells (DMSO in (+) FK, Figure 3) was significantly larger than that in DMSO in the absence of forskolin ((−) FK; ^§^ *P* < 0.001 compared with DMSO in (−) FK, Figure 3), indicating that forskolin increased the NPPB-sensitive Isc. Under forskolin-stimulated conditions ((+) FK, Figure 3), the NPPB-sensitive Isc in quercetin-treated cells was significantly smaller than that in DMSO-treated cells (^#^ *P* < 0.001 compared with DMSO in (+) FK, Figure 3), indicating that quercetin decreased the forskolin-stimulated NPPB-sensitive Isc. Contrasting the quercetin results, under forskolin-stimulated conditions ((+) FK, Figure 3), the NPPB-sensitive Isc in myricetin-treated cells was significantly larger than that in DMSO-treated cells (^##^ *P* < 0.001 compared with DMSO in (+) FK, Figure 3), indicating that myricetin increased the forskolin-stimulated NPPB-sensitive Isc. Thus, myricetin induced an NPPB-sensitive Isc at the same level irrespective of prior forskolin stimulation of Isc (1.10 ± 0.12 *μ*A/cm^2^ in the presence of forskolin, 1.16 ± 0.12 *μ*A/cm^2^ in the absence of forskolin; no significant difference). Therefore, myricetin had a synergistic effect on forskolin-activated Cl^−^ secretion. Under forskolin-stimulated conditions, quercetin did not stimulate, but rather diminished the NPPB-sensitive Isc (Quercetin versus DMSO in (+) FK, Figure 3; ^#^ *P* < 0.001) identical to that under basal conditions (Quercetin in (−) FK, Figure 3; no significant difference (NS) between Quercetin in (−) FK and (+) FK, Figure 3). In other words, forskolin had no effects on the NPPB-sensitive Isc in the presence of quercetin (compare Quercetin in (+) FK with Quercetin in (−) FK, Figure 4; NS); that is, quercetin abolished the action of forskolin on the NPPB-sensitive Isc.

### 3.4. NPPB-Sensitive Conductance under Basal and Forskolin-Stimulated Conditions

Figure 4 shows the NPPB-sensitive conductance obtained by application of NPPB (100 *μ*M) to the apical solution as described in the method. Under basal conditions, quercetin increased the NPPB-sensitive conductance (*P < 0.001 compared with DMSO in (−) FK, Figure 4) and myricetin also increased the NPPB-sensitive Gt (**P < 0.001 compared with DMSO in (−) FK, Figure 4). However, the quercetin-stimulated NPPB-sensitive conductance (37.00 ± 2.69 *μ*S/cm^2^) was slightly smaller than that stimulated by myricetin (53.00 ± 4.73 *μ*S/cm^2^; *P* < 0.05, Figure 4). The NPPB-sensitive conductance in forskolin-treated cells (DMSO in (+) FK, Figure 4) was much larger than that in DMSO in the absence of forskolin ((−) FK; ^§^ *P* < 0.005 compared with DMSO in (−) FK, Figure 4), meaning that forskolin increased the NPPB-sensitive conductance. Under forskolin-stimulated conditions ((+) FK, Figure 4), the NPPB-sensitive conductance in quercetin-treated cells was slightly but significantly larger than that in DMSO-treated cells (^#^ *P* < 0.05 compared with DMSO in (+) FK, Figure 4), indicating that quercetin increased the forskolin-stimulated NPPB-sensitive conductance. Under forskolin-stimulated conditions ((+) FK, Figure 4), the NPPB-sensitive conductance in myricetin-treated cells was much larger than that in DMSO-treated cells (^##^ *P* < 0.001 compared with DMSO in (+) FK, Figure 4), indicating that myricetin increased the forskolin-stimulated NPPB-sensitive conductance. Thus, unlike the NPPB-sensitive Isc under the forskolin-stimulated conditions, both quercetin and myricetin increased the NPPB-sensitive conductance under the forskolin-stimulated conditions (^#^ *P* < 0.05  and^  ##^ *P* < 0.001 compared with DMSO in (+) FK, Figure 4), although myricetin had much larger effects on the NPPB-sensitive conductance than quercetin (275.83 ± 15.72 *μ*S/cm^2^ in the presence of myricetin versus 130.50 ± 10.01 *μ*S/cm^2^ in the presence of quercetin under forskolin-stimulated conditions; *P* < 0.001). Further, interestingly the NPPB-sensitive Isc in the presence of quercetin was not affected by application of forskolin (compare Quercetin in (−) FK with Quercetin in (+) FK as in Figure 3; NS), but the NPPB-sensitive conductance in the presence of quercetin was much larger under forskolin-stimulated condition than that under basal conditions (compare Quercetin in (−) FK with Quercetin in (+) FK as in Figure 4; *P* < 0.001) like myricetin (see Myricetin in (−) FK with Myricetin in (+) FK as in Figure 4; *P* < 0.001). In other words, forskolin increased the NPPB-sensitive conductance in the quercetin-treated cells (compare Quercetin in (+) FK with Quercetin in (−) FK as in Figure 4; *P* < 0.001); nevertheless, forskolin had no effects on the NPPB-sensitive Isc in the quercetin-treated cells (compare Quercetin in (+) FK with Quercetin in (−) FK as in Figure 3; NS).

## 4. Discussion

Epithelial Cl^−^ secretion plays an important role in prevention from bacterial/viral infection through washout of bacteria and viruses located on the surface of apical membrane using water covering the surface of apical membrane produced by Cl^−^-secretion-generated osmotic gradient across the epithelial cells and also controls body fluid content by regulating water contents [2, 4, 7, 25–30]. The present study indicates that quercetin is useful for moderate stimulation of epithelial Cl^−^ secretion irrespective of cAMP stimulation and that myricetin can be applied in cases requiring further stimulation of epithelial Cl^−^ secretion with insufficient cAMP stimulation. Various types of flavonoids have been reported to modulate epithelial Cl^−^ secretion [9, 31–35]. For example, genistein stimulates jejunal Cl^−^ secretion via estrogen receptor-mediated pathways [31, 32]. Chao and Hamilton [9] have reported that genistein stimulates jejunal Cl^−^ secretion via phosphodiesterase modulation. Further, Fischer and Illek [34] have indicated that trimethoxyflavone, aryl hydrocarbon receptor ligand, activates CFTR Cl^−^ channel stimulating Cl^−^ secretion in lung epithelial cells. Quercetin is also reported to have stimulatory action on Cl^−^ secretion in sinonasal epithelium [35]. Niisato et al. [7] have also reported that flavonoids, genistein, daidzein, and apigenin, stimulate epithelial Cl^−^ secretion.

Although molecular mechanisms of flavonoids' action on epithelial Cl^−^ secretion are still unclear, we have a consensus that flavonoids stimulate epithelial Cl^−^ secretion via activation of the CFTR Cl^−^ channel playing a role in a Cl^−^-releasing step across the apical membrane and/or Cl^−^ transporter playing a role in a Cl^−^ uptake step across the basolateral membrane [6, 7]. Continuous activation of Cl^−^ uptake transporter such as NKCC is required to continuously stimulate epithelial Cl^−^ secretion [6, 7]. This continuous activation of Cl^−^ uptake transporter such as NKCC is one of the most important targets from a therapeutic viewpoint for continuous stimulation of epithelial Cl^−^ secretion. The present study and our previous report [3] suggest that quercetin continuously increases activity of NKCC by stimulating translocation of NKCC activating factors to the basolateral membrane from intracellular store sites.

There is little mechanistic information on the effects of flavonoids on the function of NKCC during cAMP-dependent Cl^−^ secretion, although many researchers have reported the stimulatory action of flavonoids on epithelial Cl^−^ secretion [9, 31–37] and the inhibitory action of cAMP-activated Cl^−^ secretion [38]. We were surprised that quercetin reduced the forskolin-stimulated Isc, but myricetin tremendously increased the forskolin-stimulated Isc. As mentioned above, the epithelial Cl^−^ secretion (the NPPB-sensitive Isc) consists of two steps: (1) the Cl^−^-entry step across the basolateral membrane via Cl^−^ transporter participating in Cl^−^ uptake into the intracellular space such as Na^+^-K^+^-2Cl^−^ cotransporter (NKCC) and (2) the Cl^−^-releasing step across the apical membrane via Cl^−^ channels such as cystic fibrosis transmembrane conductance regulator (CFTR) Cl^−^ channel at the apical membrane [4]. Therefore, the stimulatory action of myricetin on the NPPB-sensitive Isc could be explained by effects of myricetin on the NPPB-sensitive conductance; that is, we suggest that myricetin would increase the epithelial Cl^−^ secretion by activating the CFTR Cl^−^ channel at the apical membrane acting as the Cl^−^-releasing step. However, the inhibitory action of quercetin on the forskolin-stimulated Isc could not be explained by effects of quercetin on the apical NPPB-sensitive conductance, since quercetin increased the NPPB-sensitive conductance (in other words, quercetin activated the Cl^−^-releasing step via CFTR Cl^−^ channel; see Figure 4). Namely, if the inhibitory action of quercetin on the forskolin-stimulated Isc is explained by effects of quercetin on the NPPB-sensitive conductance (the Cl^−^-releasing step), quercetin should diminish the NPPB-sensitive conductance. Therefore, we should consider other possibilities regarding the inhibitory action of quercetin on the forskolin-stimulated Isc. There are, at least, two possible sites of action that quercetin might have on the cAMP-dependent Cl^−^ secretory process and that is either by down regulating the Na^+^-K^+^-2Cl^−^ cotransporter and/or the Na^+^, K^+^-ATPase. Similar to our data, Schuier et al. [39] have reported that quercetin, morin, and luteolin, all administered at 100 *μ*M, reduce forskolin-stimulated Isc of T84 colonic epithelial cells. These authors have offered no explanation for the action of these flavonoids. However, Collins et al. [33] have demonstrated that the flavone, naringenin, added prior to forskolin, reduces the forskolin-activated Isc of human and rat colons. They have surmised that since that action of naringenin is upstream of the activation of CFTR, naringenin inhibits NKCC. Alternatively, the action of quercetin on the forskolin-stimulated Isc might be by downregulating the Na^+^, K^+^-ATPase. Indeed, Mezesova et al. [40] have reported that treatment with quercetin (20 mg · kg^−1^ · day^−1^) in both normotensive and hypertensive rats produces impairment in the affinity of Na^+^ binding site of the Na^+^, K^+^-ATPase like the inhibitory action on Ca^2+^-ATPase [41, 42]. On the other hand, myricetin showed no inhibitory action but stimulatory action on Isc, suggesting that myricetin would not inhibit NKCC or produce impairment in the affinity of Na^+^ binding site of the Na^+^, K^+^-ATPase unlike quercetin. Further work is necessary to resolve this complex role of quercetin in cAMP-dependent Cl^−^ secretion.

Although it has been reported that flavonoids including myricetin and quercetin have various action on cell function [3, 10, 31–33, 43–51], the present study clearly indicates that myricetin has stimulatory action on cAMP-activated Cl^−^ secretion unlike quercetin. From our knowledge, the present study is the first report showing a flavonoid with stimulatory action on cAMP-activated epithelial Cl^−^ secretion. Thus, using these compounds it could be possible to treat patients with disorders in water secretion across epithelial tissues by regulating epithelial Cl^−^ secretion to ideal levels depending on pathophysiological states of patient.
